# Harnessing thermally treated drinking water sludge: a sustainable approach for the removal of crystal violet and congo red from wastewater

**DOI:** 10.1038/s41598-025-02272-9

**Published:** 2025-05-20

**Authors:** Doaa A. El-Emam, Amany H. Elezaby, Mohammed A. Zeyadah, Mervat A. El-Sonbati

**Affiliations:** Environmental Science Department, Faculty of Science, Damietta University, New Damietta, Egypt

**Keywords:** Circular economy, Environmental sustainability, Global challenges, Sustainable waste management practices, Wastewater treatment, Ecology, Environmental sciences

## Abstract

This study investigates the utilization of thermally treated drinking water treatment sludge (DWTS) as an eco-friendly adsorbent for the removal of Congo Red (CR) and Crystal Violet (CV) dyes from wastewater, aligning with circular economy principles. The research evaluates the adsorption performance of DWTS by analyzing various factors, including pH, contact time, adsorbent dosage and initial dye concentration. Kinetic and isotherm studies were conducted to elucidate the performance of the adsorbent and investigate the adsorption mass transfer mechanisms. Characterization of the DWTS adsorbent was performed using Energy Dispersive Spectrometry (EDS), Scanning Electron Microscopy (SEM), Zeta potential, point of zero charge, Brunauer–Emmett–Teller (BET), and Fourier Transform Infrared Spectroscopy (FTIR) analysis. The results revealed that the optimal conditions for dye removal were established at 120 min, 2 g adsorbent dosage, 50 mg/L dye concentration, pH5 and pH9, achieving 94.3% and 86.9% for CR and CV, respectively. The dye adsorption equilibrium data fitted well to the Langmuir isotherm model with monolayer maximum adsorption capacity of 21.368 and 10.1419 mg/g for CR and CV dye, respectively. In addition, the kinetic studies showed rapid sorption dynamics following a First-order kinetic model. Moreover, the intra particle diffusion and Elovich models exhibited high correlation coefficient values indicating a contribution of physical and chemical adsorption process. These findings suggest that DWTS is a cost-effective and viable alternative for dye removal in wastewater treatment, with implications for sustainable waste management practices. Additionally, recommendations for the safe disposal of spent adsorbents are discussed, highlighting potential applications in construction materials.

## Introduction

Climate Changes, water scarcity and water pollution have become global challenges, exacerbated by rapid urbanization, industrialization, and technological advancements^1^. Given the present spending rate, this scenario will only deteriorate. By 2025, two-thirds of the global population may see water scarcity. In addition, ecosystems around the world will suffer even more^2^. To address these challenges, it is crucial to focus on removing harmful contaminants from water sources. Among the numerous contaminants, dyes and pigments constitute a significant category due to their extensive application in sectors such as paint, paper, and textiles.

Many dyes are carcinogenic, toxic and can have detrimental effects on both the environment and human health, making their removal a top priority in water treatment processes^3^. Dyes cause dermatitis, vomiting, jaundice, nausea, mutations, allergies, and heart defects in addition to being harmful to both people and microorganisms so, the aquatic systems may sustain damage if these dyes are released into the water at different rates^4^. Approximately 10% of dyes utilized in the textile sector are wasted during the dyeing process, and 2% are directly released as aqueous effluents into the environment without sufficient treatment. These compounds maintain their structural integrity and colouration when exposed to soil, sunshine, bacteria, and perspiration, demonstrating significant resistance to microbial degradation during the wastewater treatment process which demands innovative solutions^5^.

Crystal violet (CV) is one of the most common cationic, synthetic dyes, belongs to triarylmethane group with the chemical formula C_25_H_30_ClN_3_ (C.I. 42555, MW: 407.98 g/mol) as shown in Table 1, is widely used in textile dyeing, paper dyeing, biological staining and paintings^6,7^. CV can result in mammalian cell toxicity, digestive tract and skin irritation, and renal and respiratory failure. Cationic dyes have much greater toxicity compared to anionic dyes^4^.

Azo dyes, exemplified by Congo Red (CR) with the molecular formula C_32_H_22_N_6_Na_2_O_6_S_2_ and a molecular weight of 696.7 g/mol (Table 1), constitute a principal category of synthetic colourants commonly employed in textile production owing to their extensive range of hues, resistance to fading, and reduced energy consumption. The Congo Red (diazo dye) is classified as a carcinogen due to the presence of an aromatic amine. Aromatic structures confer resistance to natural degradation in azodyes^8,9^.

**Table 1:** Properties of crystal Violet and congo red dyes^10,11^.

Organic dyes	Molecular formula	Molecular structure	Molecular weight (g/mol)
CV	C_25_H_30_ClN_3_		407.99
CR	C_32_H_22_N_6_Na_2_O_6_S_2_		696.7

Each year, various types of dyes are manufactured globally, totalling up to 108 tonnes, with azo dyes constituting 60–70% of this amount. The extensive use of azo dyes results in a significant volume of wastewater containing azo dye contaminants^12^.

Several biological, physical, and chemical treatment techniques have been made for the removal of dyes from contaminated wastewater such as advanced oxidation, electrochemical degradation, ion exchange, aerobic, and anaerobic digestion, bioremediation, adsorption, photodegradation, membrane filtration, combined chemical and biochemical processes^13–20^.

Actually, the toxicity and recalcitrant nature of the synthetic dyes render the application of traditional biological techniques for their removal^21^. Moreover, Physical treatment methods can only remove floating or scattered pollutants and are economically unfeasible due to the requirement for costly materials and the extensive time needed for integrated procedures. Moreover, the creation of secondary pollutants poses significant environmental challenges^22–24^.

Among the remediation methods, adsorption stands out among these techniques because of ease of use, simple operating design, economic feasibility, resistance to contaminants, environmental friendliness, low cost, availability, sustainability, and large adsorption capacity^25^. Moreover, it is considered one of the most ecologically friendly approach for color removal^26,27^.

The ongoing quest for novel eco-friendly adsorbents that exhibit enhanced efficiency and reduced cost is a pressing requirement in the after-treatment sector. Drinking Water Treatment Sludge (DWTS) is particularly significant in numerous countries, including Egypt, where it is estimated that approximately 15% of the population utilises surface water. The annual production of wet sludge is around 1,500,000 tonnes, which decreases to 750,000 tonnes per year after dewatering. DWTS comprises colloidal iron and aluminum hydroxides, with aluminum and iron being the predominant coagulants, alongside colloidal or dissolved organic materials, clay, silt, and microorganisms. They are frequently referred to as hydroxide sludge due to the presence of metal hydroxides^28^.

This residual or sludge is normally incinerated or disposed in landfill after biological or chemical stabilization processes. To adhere to the principles of the circular economy and minimise the establishment of new landfill sites, alternative options for managing this residual are essential^29^. The principle of utilizing waste materials for value creation has garnered growing interest from both environmental organisations and scientists.

This study aims to bridge the existing research gap by utilizing thermally treated Drinking Water Treatment Sludge (DWTS) as an innovative, eco-friendly adsorbent for the removal of CV and CR dyes from wastewater. Key parameters influencing the adsorption process, including contact time, the dye’s pH, adsorbent dosage, and initial dye concentration will be critically evaluated. Additionally, adsorption kinetics, isotherms, and underlying mechanisms will be examined to assess the effectiveness of DWTS for dye uptak. Through such exploration, this research endeavors to contribute pivotal strategies for effective dye waste management while aligning with circular economy principles to minimize environmental impact.

## Materials and methods

### Reagent and chemicals

The chemicals employed in this investigation are of analytical grade and were utilized without any purification. Congo Red (C_32_H_22_N_6_Na_2_O_6_S_2_), Hydrochloric acid (HCL), Sodium hydroxide (NaOH), and Crystal Violet (C_25_H_30_ClN_3_) were purchased from the Egyptian Chemical Industries Company. DWTS was obtained from the Damietta power station, Damietta, Egypt.

### Preparation of DWTS adsorbent

**Fig. 1:**
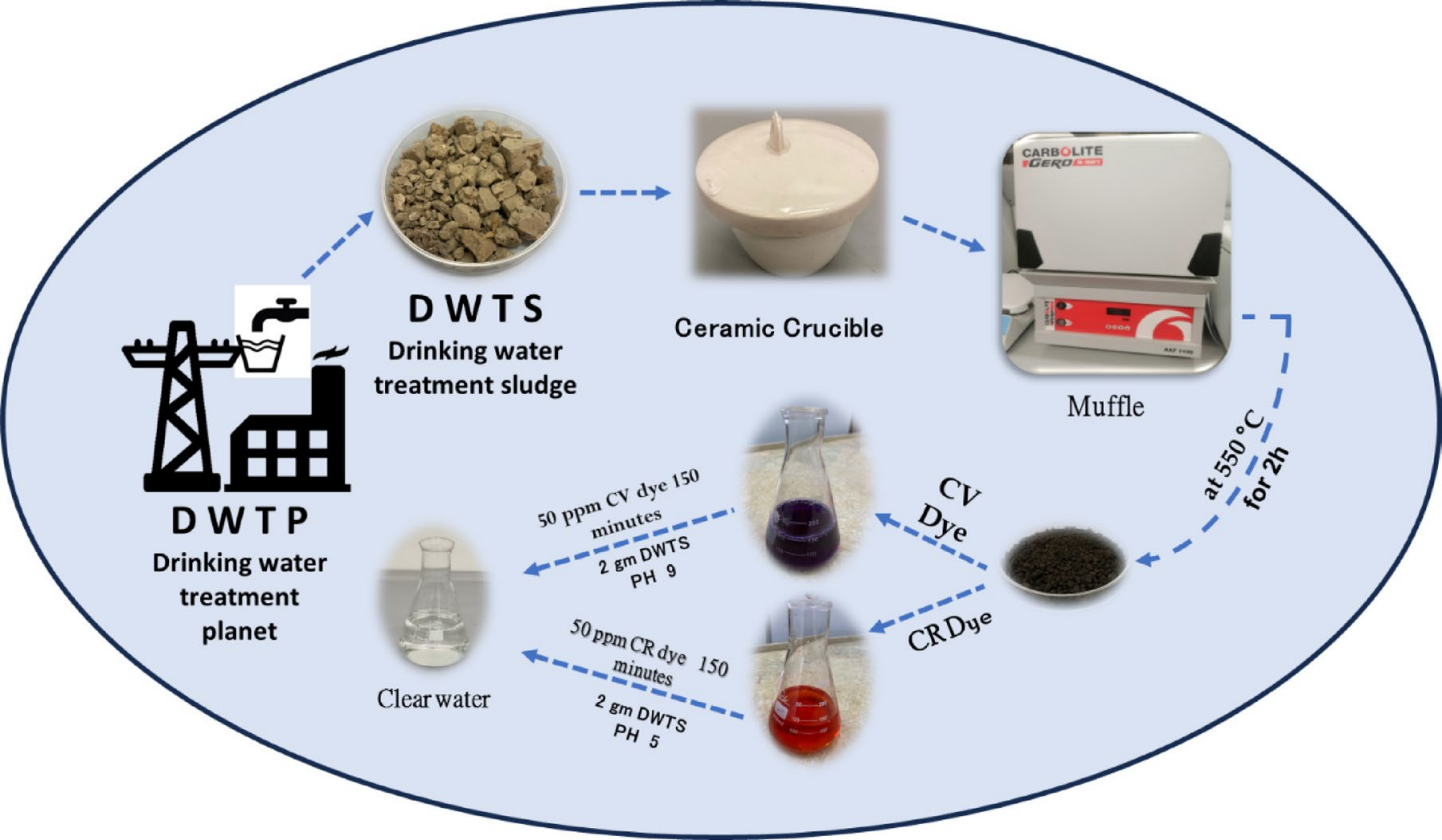
Schematic diagram for preparation of DWTS adsorbent and removal of CV, CR dyes from wastewater solutions.

The Drinking Water Treatment Sludge samples were collected from Damietta power station (Egypt) which has a drinking water production facility inside the site, from Nile River water. The collected DWTS was desiccated in an oven at 105 °C for 30 min, thereafter diced into small fragments, pulverised into a fine powder, and sifted through a 1 mm mesh. Subsequently, 1.0 g of sieved fine powder was subjected to pyrolysis in a porcelain crucible within a muffle furnace at temperatures of 300 °C, 350 °C, 400 °C, 450 °C, 500 °C, and 550 °C for a duration of 2 h, employing a heating rate of 10 °C/min (Fig. 1).

### Characterization of the DWTS adsorbent

All characterization was conducted at the National Research Center in Cairo City. Chemical and morphological structure of the DWTS adsorbent were analyzed using various devices. Before conducting scanning electron microscopy (SEM) examination, samples were attached to aluminum microscope stubs using adhesive carbon tape. A Quorum Technologies Ltd sputter coater (Q150T, England) was used to coat the samples with gold (Au) for 120 s. The analysis was carried out using a Tescan SEM (TESCAN VEGA 3, Czech Republic).

The elemental composition of the as-prepared adsorbent was determined by Energy Dispersive Spectrometry (EDS), on a NOVA touch 4LX (St 2; s/n:17016062702]). A Shimadzu FT-IR 8400 S spectrometer was used for the Fourier Transform Infrared Spectroscopy (FT-IR), Infrared (IR) spectra were generated using the dry KBr disc method and covered a range from 400 to 4000 cm^−1^.

The Brunauer–Emmett–Teller(BET) method was utilized to examine the impact of thermal treatment onthe specific surface area and pore structure of DWTS powder. The adsorption-desorption isotherm of N_2_ was analyzed using Instrument St 2 on NOVA touch 4LX [s/n:17016062702]. The samples’ pH values were measured with an ADWA digital pH meter (ADWA 1000 CE, Romania). The point of zero charge pH (pH_pzc_) was ascertained as follows: One gram of the DWTS adsorbent was introduced into 40.0 mL of 0.1 M NaNO3 across ten 120 mL bottles. The pH was adjusted from 1 to 13 utilizing 0.1 M HCl and 0.1 M NaOH, and the mixture was shaken for 24 h to reach equilibrium. Subsequently, the final pH (pHf) was assessed, and a graph was constructed depicting the starting pH (pHi) in relation to the difference between pHi and pHf (ΔpH). The pH_PZC_ was derived from the intersection of the graph with the X-axis. On the other hand, Zeta potential was analyzed by a Zetasizer Nano2000 (Malvern Instruments, UK).

### Optimization of DWTS adsorbent performance

The optimal adsorption conditions (pH, dye concentration, contact time, and adsorbent dose) for CV and CR dyes on the DWTS adsorbent were determined in a batch mode, stock solutions of each dye were prepared and subsequently diluted with distilled water to create working solutions. The dye concentrations were quantified using a single beam spectrophotometer (PG T60 UV/VIS Spectrophotometer, PG Instrument Ltd, UK) using quartz cells with a route length of 10 mm. The pH of the dye solutions was precisely adjusted to the correct level using 0.1 M NaOH and HCl solutions.

To evaluate the adsorption efficacy, 2 g of DWTS adsorbent was added to 50 mL of the dye solution (50 mg/L concentration) and agitated with a lab-line Stuart Scientific Orbital mechanical shaker (SO1, UK) at 25 °C for various mixing durations. The adsorbent was then extracted from the aqueous solution, as well as the absorbance of the (CV and CR) dye solutions was measured at (585 and 498 nm). The influence of several parameters, including contact duration (10, 30, 60, 90, 120, and 150 min), pH (3, 5, 7, 9, and 11), starting dye concentrations (50–250 mg/L), and adsorbent dosages (0.1–2 g), on the adsorption process was thoroughly examined.

The adsorption capacity (mg/g) of the DWTS adsorbent for CV and CR dyes was calculated^30,31^ using the following equation:

1 $$Q = \left( {C_{o} - C_{e} } \right)*V/W$$

where V, W, C_o_ and C_e_ represent the volume (L) of the solution, weight (g) of DWTS, the initial and equilibrium concentrations (mg/L), respectively.

The dye removal efficiency is calculated^32^ as follows:

2 $$R\% = \left( {Ci{-}Ce} \right)/Ci*100$$

where C_e_ and C_*i*_ are the final and initial concentrations of CV and CR (mg/l).

### Adsorption isotherms

Adsorption isotherms were conducted by agitating various initial CV and CR dye concentrations (50 to 250 ppm) with 2 g of DWTS adsorbent for 150 and 120 min, respectively at the optimum pH for each dye. Following the establishment of equilibrium, the quantity of adsorbed dye (qe, mg/g) was quantified and graphed in relation to the equilibrium concentration (Ce, mg/L). Freundlich, Temkin, and Langmuir models were employed to display the well fit with the adsorption process^33,34^.

### Adsorption kinetics

A kinetic investigation was conducted by stirring 50 mL of each CV and CR dye (50 ppm) individually with 2 g of DWTS adsorbent for various time intervals (10–150 min). The dye concentration was assessed at each time interval, and a plot of the adsorbed dye amount (qt, mg/g) vs. time (t, min) was generated for kinetic modelling^34^.

## Results and discussions

### Characterization of the DWTS adsorbent

#### Morphological structure identification SEM

**Fig. 2:**
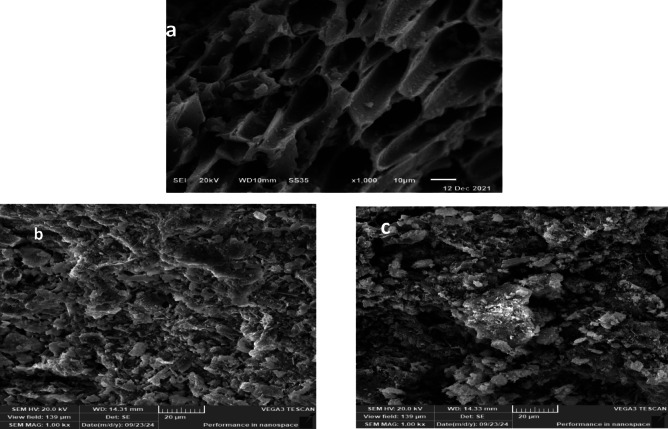
SEM of (**a**) DWTS adsorbent, (**b**) and (**c**) CV and CR loaded DWTS adsorbent at x1000.

Figure 2 shows the surface morphology of the DWTS adsorbent prior to and following exposure to the dye models CV and CR^35^. It is clear that, there were many cylindrical voids on the DWTS adsorbent surface^36^, that might enhance its adsorption capabilities; which disappeared due to the coverage of the dye’s molecules and adsorption^37^ as shown in Fig. 2b and c.

#### Energy dispersive spectrometry (EDS)

The elemental composition of DWTS was confirmed by EDS. The deposition of key elements Fe, O_2_, C, Al, Si and Ca on sorbents surfaces was evidenced in Table 2. It was affirmed that the existing of Fe, O_2_, C, Al, Si and Ca elements within % (44.01: 16.86: 7.32: 3.10: 1.61: 0.63) for DWTS^35^.

**Table 2:** The elemental composition of DWTS adsorbent by EDS analysis.

Element	At. no.	Mass (%)	Mass norm. (%)	Atom (%)
Iron	26	44.01	59.86	29.86
Oxygen	8	16.86	22.94	39.94
Carbon	6	7.32	9.95	23.08
Aluminum	13	3.10	4.21	4.35
Silicon	14	1.61	2.19	2.17
Calcium	20	0.63	0.85	0.59
		Sum 73.52	100.00	100.00

#### Identificationof chemical structure via fourier transform infrared (FTIR) spectroscopy

**Fig. 3:**
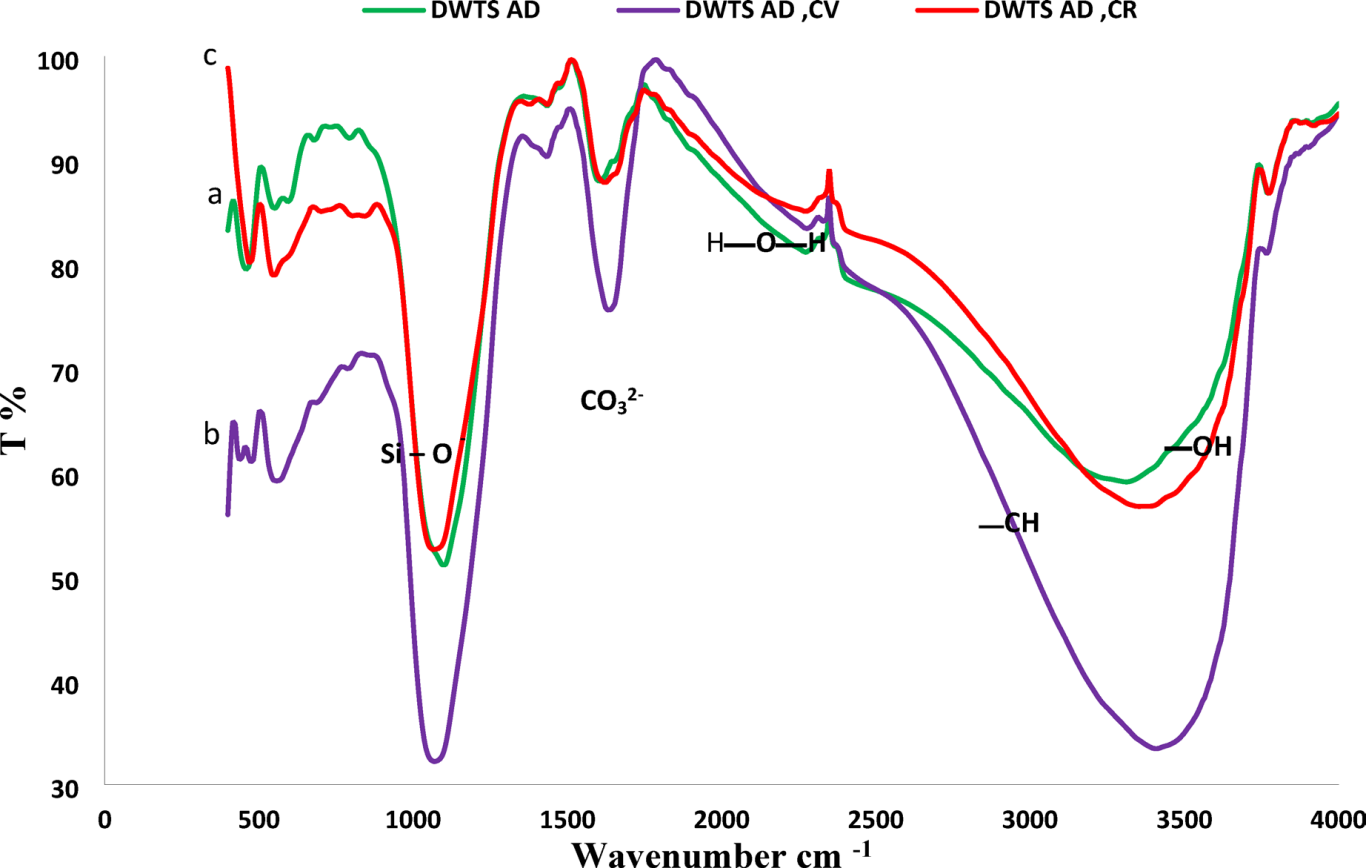
FTIR (**a**) DWTS adsorbent, (**b**) and (**c**) CV and CR loaded DWTS adsorbent.

FTIR analysis was conducted to identify the distinctive functional groups of DWTS, as presented in Table 3 and Fig. 3, before to and following the adsorption of both CV and CR. The recorded spectra indicate a broad band at 3458.71 cm ^−1^, often attributed to the valence vibration of the hydroxyl group O–H in the adsorbed water molecules^38^, where the sharp peaks at 2859.92 cm^−1^ and 2925.48 cm^−1^ are corresponding to − CH_2_ stretching. The band performed at 1665.23 cm^−1^ is attributed to the angular deformation of H–O–H in water molecules^39^. The existence of calcite CaCO_3_ in sludge is validated by the band of CO_3_^− 2^ at 1440.56 cm^−1^^36^. The intensive bands at 1000–1100 cm^−1^ were linked to Si–O–Si stretching vibrations, indicating the presence of silanol groups, while peaks at ~ 500–700 cm^−1^ were related to metal oxides such as Al–O and Fe–O, indicating the hydroxide composition of DWTS^35^. Furthermore, the band at 540.935 cm^−1^ is associated with coupled Al–O and Si–O vibrations^37^. The shoulder at about 471.51 cm^−1^ is ascribed to the deformation vibrations of the symmetrical Si–O–Si bonds and the asymmetric Si–O–Al bonds^40^.

Comparing FTIR after adsorption of CR and CV dyes, significant changes were observed in the spectra as displayed in Table 3 and Fig. 3. For CV, a cationic dye, the peaks associated with negatively charged groups such as COO⁻ and Si–O–Si were reduced in intensity or disappeared entirely, indicating their active involvement in electrostatic interactions with the positively charged CV molecules where, new peaks at ~ 1500–1600 cm^−1^ suggested the formation of π-π interactions between the aromatic rings of CV and the surface functional groups of DWTS. Due to anionic dye CR, the O-H and C = O peaks were much weaker than expected, which may indicate electrostatic interactions and hydrogen bonding between CR’s sulfonate groups and the protonated functional groups on DWTS. Additional peaks at ~ 700–900 cm^−1^ indicated specific vibrational modes arising from the attachment of CR molecules to the adsorbent surface^41,42^. These spectral changes confirm the participation of functional groups in the adsorption of both CV and CR, with the nature of interactions differing based on the molecular structure and charge of the dyes^34^.

**Table 3:** Infrared spectra of DWTS adsorbent, before and after adsorption of CV, CR dyes.

	−OH cm^−1^	−CH cm^−1^	H−O–H cm^−1^	CO_3_^2−^ cm^−1^	Si–O^−^cm^−1^	Al–O, Si–O cm^−1^	Si–O–Si, Si–O–Al
DWTS	3458.71	2859.92-2925.48	1665.23	1440.56	1041–1092	540.935	471.51
DWTS /CV	3407.6	disappear	1623.77	1,435,074	1069	478.26	437.76
DWTS / CR	3355.53	disappear	1636.3	1473.35	1066	547.68	472.47

#### The Brunauer–Emmett–Teller BET

**Fig. 4:**
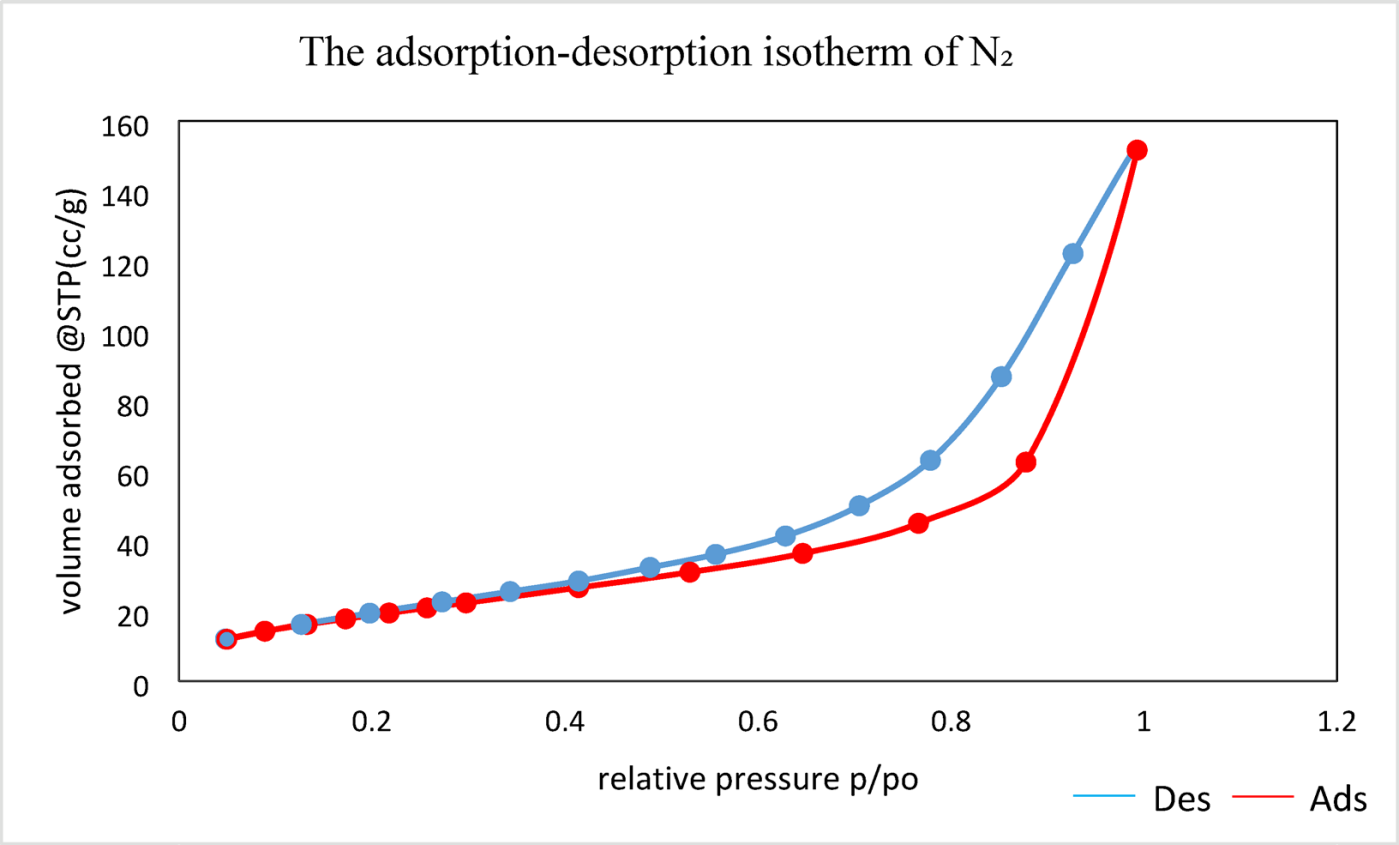
The adsorption-desorption isotherm of N_2_ for DWTS adsorbent.

In order to study the structure of the DWTS adsorbent, a standard N_2_ adsorption-desorption isotherm was recorded. Figure 4 clearly shows that the sorption isotherms comply with the IUPAC categorization of mesoporous materials, specifically type IV. Mesopores may be responsible for the presence of an H_2_-type hysteresis loop in the p/p0 relative pressure range of 0.4139 to 0.92. Also, for relatively high pressures between 0.92 and 1.00, there was no plateau, which could mean an H_3_-type hysteresis loop and the formation of particle agglomerates^37^. The specific surface area measured was 41.5458 m²/g, with a total pore volume of 2.3519e-001 cc/g and a predominant pore size of 1.91945 nm, all determined at a relative pressure of 0.99290. As a result, the texture of DWTS becomes increases in density as substantial pore closure occurs, demonstrated the existence of numerous active sites conducive to adsorption. Thus, the increased surface area and reduced pore volume render DWTS suitable and promising for the adsorption of CR and CV dyes^43^.

Post-adsorption analysis revealed notable reductions in the pore volume and specific surface area. As adsorption of CV led to a significant decrease in mesoporous volume, likely due to the larger molecular size of CV, which occupied surface sites and blocked access to internal pores. While in CR adsorption process, the reduction in microporous volume was more pronounced, reflecting the ability of the smaller CR molecules to penetrate and adsorb within smaller pores. Additionally, the hysteresis loop observed in the nitrogen adsorption-desorption isotherm indicated a shift in pore structure post-adsorption, suggesting a partial collapse or clogging of pores by the adsorbed dyes, the effect was more pronounced for CV, indicating the greater impact on the textural properties of DWTS.

#### Point of zero charge (pzc) and zeta potential

**Fig. 5:**
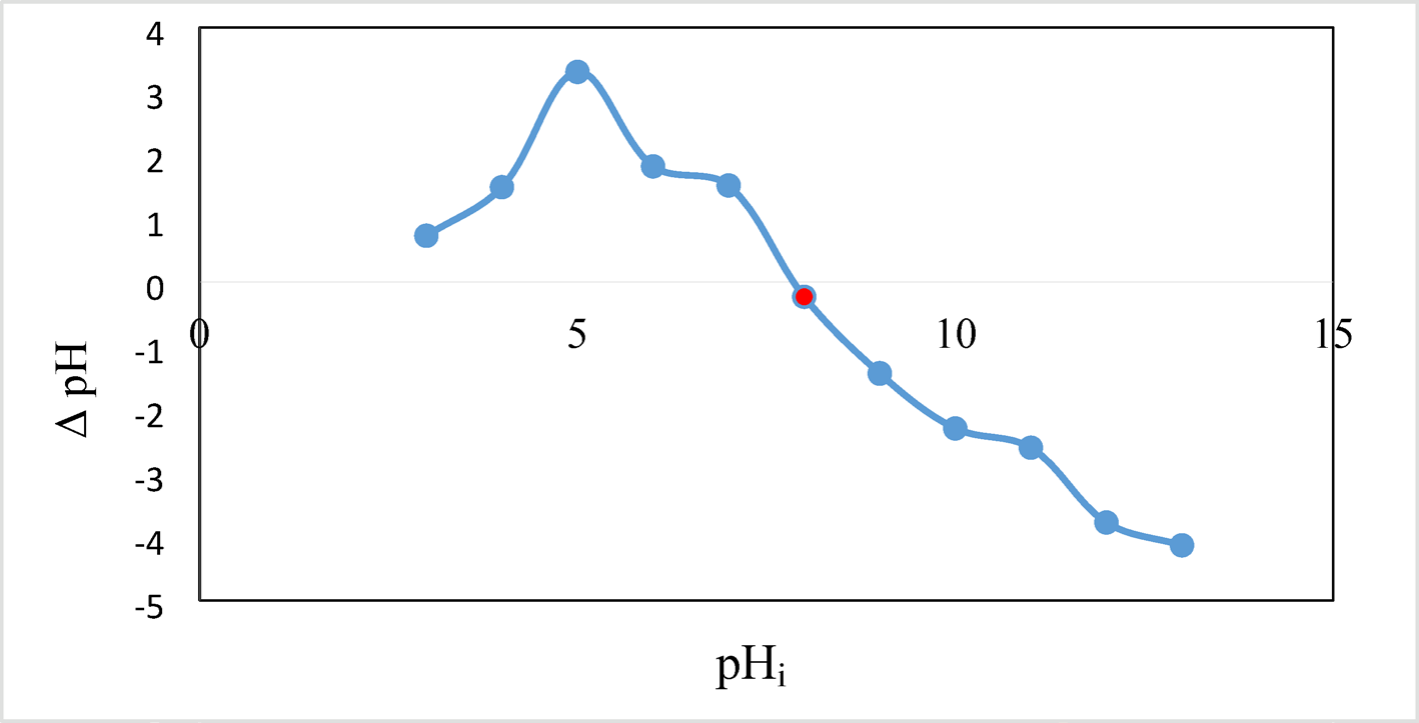
The analyzing the PZC of DWTS.

pHpzc is significant in surface science as it reflects how readily a substrate can adsorb potentially harmful ions^44^. The pzc of the DWTS was investigated, and the result (7.8) was presented in Fig. 5. Thus, at pH 7.8 surface of the adsorbent reaches electrically neutral state while below which, the surface carries a positive charge, enhancing anionic (CR) adsorption, and above which, the surface charge becomes negative, preferring cationic (CV) adsorption. Consequently, the removal of CV and CR dyes by DWTS adsorbent is favored at pH of 5 and 9, respectively, as the pH_pzc_ is affected by the functional groups that are present on the surface^45^.

**Fig. 6:**
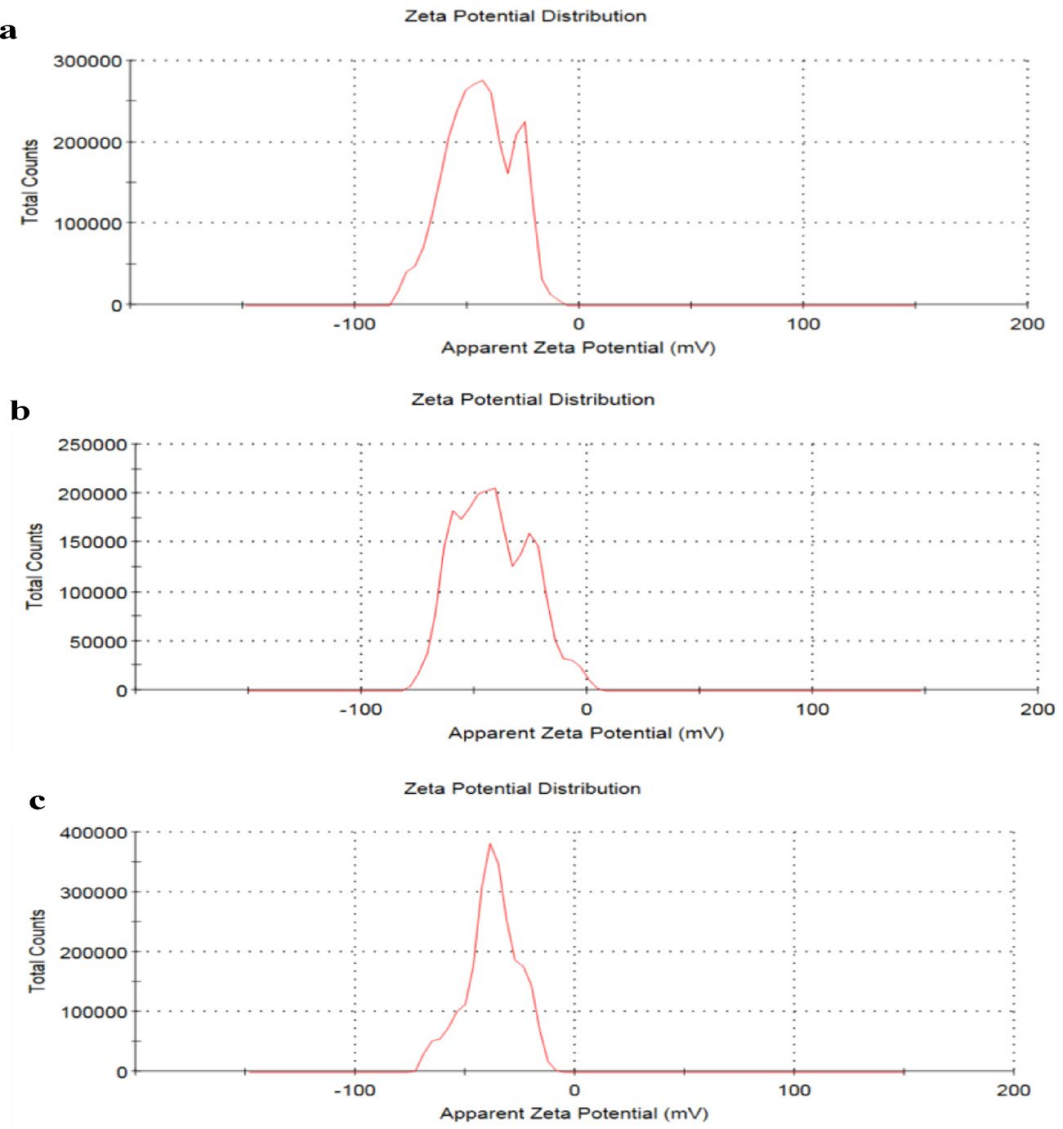
The analyzing zeta potential of DWTS, (**a**) zeta potential = -44.5 mV; (**b**) zeta potential =-41.8 mV; (**c**) zeta potential =-37.8 mV.

It is clear from Fig. 6 that zeta potential value is -41.3 ± 3.37) mV, indicating an electrostatic interaction between the investigated dyes and DWTS surfaces which significantly influences adsorption capacity^20,23^.

### Performance of DWTS adsorbent

The capacity of the thermally modified DWTS to adsorb CV and CR dyes from aqueous solutions was assessed using batch mode experiments conducted at room temperature. The impact of different operational parameters on the elimination of CV and CR dyes was thoroughly examined.

#### Effect of contact time

**Fig. 7:**
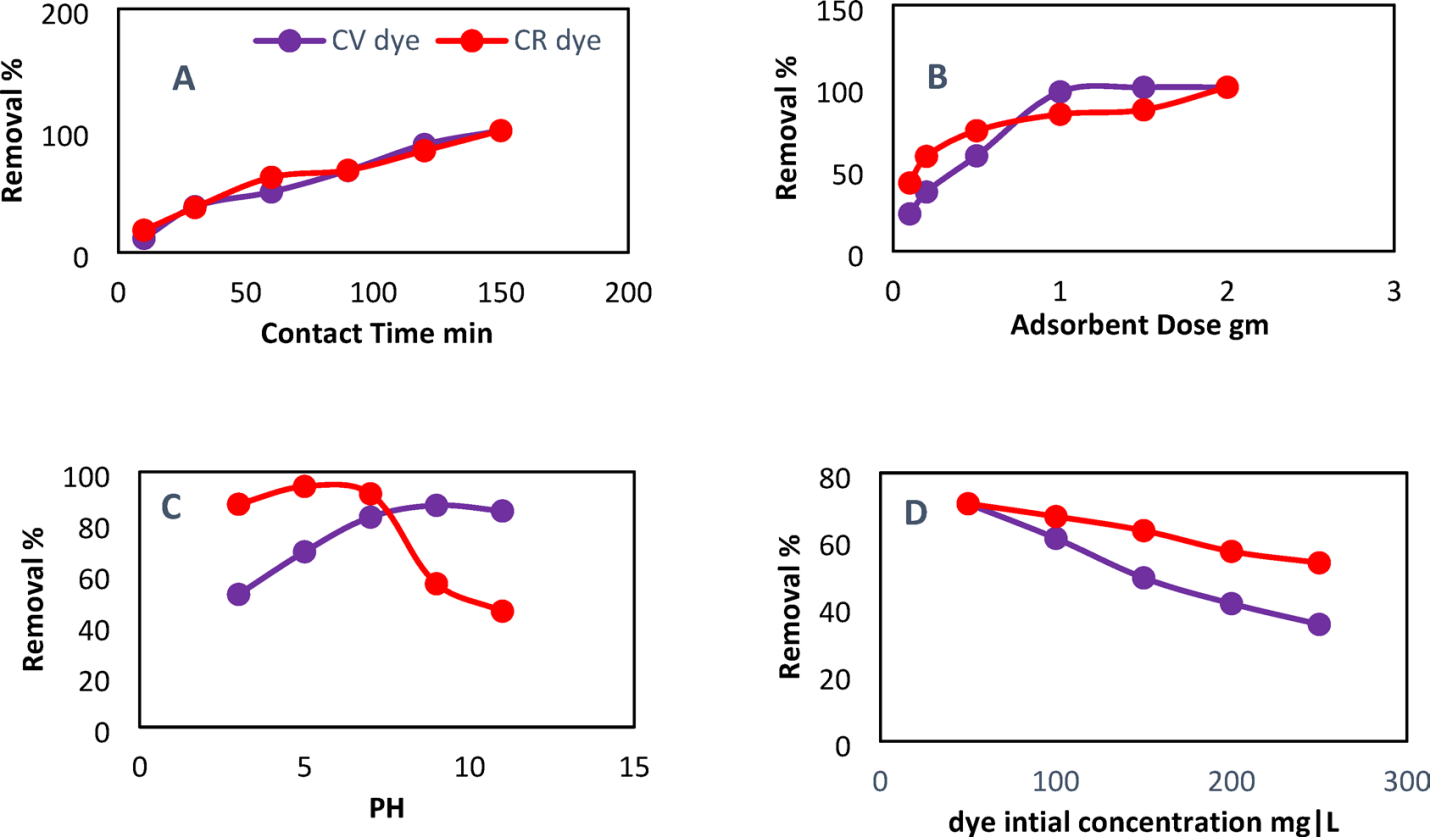
Effect of different operational parameters on the adsorption of CV, CR dyes (50 mL) by the thermally modified DWTS (**A**) Contact time (min), (**B**) Adsorbent Dose (gm), (**C**) pH and (**D**) Initial dye concentration (mg/L) at 25 °C.

The investigation focused on the impact of contact time (10–150 min) on the adsorption of CV and CR dyes (50 ml of 50 mg/l) by the DWTS adsorbent. Typically, the percentages of removal rise as contact time extends, attributed to the improved capacity for adsorbate uptake on the surfaces of adsorbents^46^. It is clear from Fig. 7A that the optimum contact time was 120 min with removal percentage 88.6% and 83.5% for the adsorption of CR, CV dyes by DWTS adsorbent, respectively. This suggests that the adsorption process occurred rapidly, facilitating the removal of the CR and CV molecules from the solution to the surface of the DWTS adsorbent. Subsequently, intermolecular interactions between the positively charged CV groups and the negatively charged functional groups may take place^47–49^.

#### Effect of adsorbent dosage

Experiments were conducted to assess the impact of varying dosages of DWTS adsorbent (ranging from 0.1 to 2 g) on dye removal at a controlled temperature of 25 °C^30^. The results presented in Fig. 7B indicate that the removal percentage of CV and CR dyes rose from 36.12 to 97.17% and from 57.82 to 83.5%, respectively, as the adsorbent dose increased from 0.2 g to 1 g. A complete uptake of 100% was achieved with an adsorbent dose of 2 g. This improvement can be attributed to the increase in surface area, the number of adsorbent pores, and the availability of active sites, which collectively enhanced dye uptake^50^.

These results are similar to that obtained by^51^ who recorded removal percentage 99.89% and 86.24% with increasing adsorbent dose to 1.5 g, for CV and CR, respectively, and 100% removal at adsorbent dose 2 gm. Consequently, a dosage of 2 g was established as the optimal choice for the following experiments.

#### Effect of pH

The pH of the adsorption process can significantly influence the surface charge of the adsorbent as well as the ionization of the adsorbate^31^. The influence of pH levels (3, 5, 7, 9, and 11) on the absorption of CV and CR dyes by DWTS was examined. The data presented in Fig. 7C indicate an increase in removal percentage with rising pH, achieving a maximum value of 86.9% at pH 9 for CV. In contrast, CR reached a maximum removal percentage of 94.34% at pH 5, followed by a decline as pH increased. This phenomenon can be attributed to the interplay between the surface charge of the adsorbent and the chemistry of the solution, both of which are critical factors. The protonation of functional groups causes cation exchange between dye molecules and hydrogen ions from the protonated groups, which causes CV, a cationic dye, to present a positively charged adsorbent surface at pH values below pH_pzc_. Sodium bicarbonate and free positive hydrogen in acidic solutions compete with dye cations (CV^+^), reducing dye uptake. Electrostatic attraction between the active sites and dye cations occurs when the pH level exceeds pH_pzc_, however, this occurs only when the adsorbent surface gains a negative charge. Conversely, the dye molecules present on the adsorbent surface could establish π-π interactions with adjacent molecules^44,46^. The contrary behaviour was observed for the anionic CR dye^31,44^. The findings were further validated by additional reported data regarding cationic and anionic dye adsorption^31,44,52^. The surface interaction of DWTS adsorbent can affect CV, CR dyes which may depend on the concentration of H^+^ ions in the solution^44,52^.

#### Effect of initial dye concentration

The impact of initial dye concentration (50–250 mg/l) on the adsorption of CV and CR by DWTS adsorbent was examined, with the resulting data presented in Fig. 7D^23^. A significant removal percentage of both dyes at low concentrations was noted, attributed to the presence of available and unoccupied active sites on the surface of DWTS^31,44,52^. Comparable behaviour was noted by^53^, who indicated that the removal efficiencies for both CV and CR (50 ml of 50 mg/L) at pyrolyzed industrial carpet waste were 76.8% and 70.83%, respectively, with a decline observed as the initial dye concentration increased.

### Kinetic studies

The application of adsorption kinetic models facilitated the prediction of the removal mechanism, rate, and efficiency of DWTS adsorbent in the elimination of CV and CR dyes^33,54^. Kinetic studies were conducted utilizing the pseudo-first order, pseudo-second-order, intra-particle diffusion, and Elovich equations. The pseudo-first-order model illustrates Lagergren’s equation for liquid-solid adsorption, accommodatingphysisorption on a homogeneous surface. This approach aids in identifying the characteristics and order of the DWTS adsorbent, which is crucial for determining the removal rate. The linear expression for Pseudo first order^55^ was displayed as:

3 $$\log \left( {Q_{e} - Q_{t} } \right) = \left( {\log Q_{e} } \right) - \left( {K_{1} t/2.303} \right)$$

**Fig. 8:**
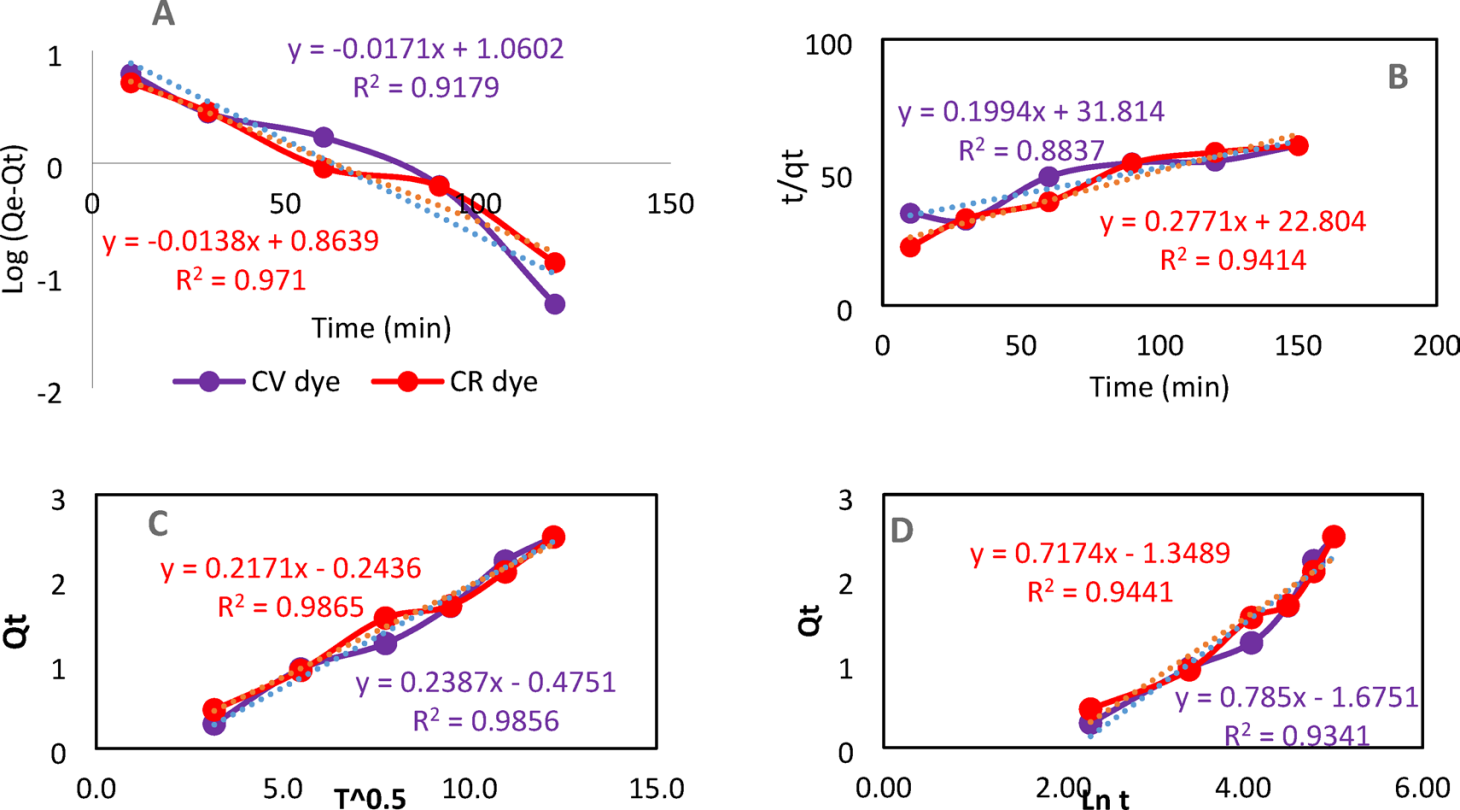
Kinetic adsorption models (**A**) pseudo first order (**B**) pseudo second order (**C**) Intra-particle Diffusion (Weber-Morris) (**D**) Elovich for removal of CV, CR dyes by DWTS adsorbent (C CV, CR = 50 mg/L, 50 mL and 2 gm adsorbent dose at 25 °C).

In this context, Qe and Qt denote the removal capacity (mg/g) at equilibrium and at time t (min), respectively, while K1 signifies the pseudo-first-order constant (L.min^−1^). The pseudo-first-order model is expressed as a function of time through log (Qe - Qt), with the constants detailed in Table 4 and illustrated in Fig. 8A. The correlation coefficient (R^2^) values recorded were 0.9179 and 0.971 for the removal of CV and CR dyes by the DWTS adsorbent, respectively. The theoretical Qe values were determined from the intercept of the plot as 11.49 and 7.309 for the adsorption of CR and CV dyes by the DWTS adsorbent, respectively. The pseudo second-order model illustrates the removal process governed by chemisorption (which may involve complexation and itraparticle diffusion. The linear form for pseudo second order^47^ was expressed by the following equation:

4 $${\raise0.7ex\hbox{$t$} \!\mathord{\left/ {\vphantom {t {Q_{t} }}}\right.\kern-\nulldelimiterspace} \!\lower0.7ex\hbox{${Q_{t} }$}} = {\raise0.7ex\hbox{$t$} \!\mathord{\left/ {\vphantom {t {Q_{e} }}}\right.\kern-\nulldelimiterspace} \!\lower0.7ex\hbox{${Q_{e} }$}} + \left( {{\raise0.7ex\hbox{$1$} \!\mathord{\left/ {\vphantom {1 {K_{2} Q_{e}^{2} }}}\right.\kern-\nulldelimiterspace} \!\lower0.7ex\hbox{${K_{2} Q_{e}^{2} }$}}} \right)$$

where Q_e_ and Q_t_ represent the removal capacity (mg/g) at equilibrium and at time t (min), respectively, while K_2_ denotes the pseudo-second-order constant (g/mg. min). The pseudo-second-order is expressed as a function of time using t/Q_t_, which is suitable for the removal process. The values of the correlation coefficient R^2^ were 0.8837 and 0.9414 for the adsorption of CV and CR dyes by the DWTS adsorbent, respectively. The rate constant (k_2_ g/mg. min) was determined from the intercept of Fig. 8B, and the results were presented in Table 4.

The diffusion rates of CR and CV dyes onto DWTS adsorbent were analyzed through the Weber-Morris and Elovich models, illustrating the stages of the adsorption process that may be influenced by external or film diffusion, pore diffusion, and adsorption on the pore surface, or a combination thereof^56^.

First film diffusion occurs when mass transfer particles of CR and CV dyes independently move from the outside surface to the active sites of DWTS adsorbent and diffusion onto the surface of the adsorbent may occur simultaneously, the second step is the transfer of the CV, CR molecule from the film diffusion to the interior sites of DWTS adsorbent while the third step includes CV, CR molecule sorption in the inner pores of DWTS adsorbent^57^.

The kinetics of intra-particle diffusion, as described by the Weber-Morris model, illustrates the influence of the boundary layer. The significance of the C value suggests that the contribution of surface adsorption will play a more substantial role in the rate-controlling step. The linear expression for the Weber-Morris model^34^ was presented as follows:

5 $$Qt = Ki\surd \left( t \right) + Ci$$

where Q_t_, k_i_, and C were the removal capacity (mg/g) at time t, diffusion rate constant (mg/g.min^0.5^), and boundary layer thickness (mg/g), respectively. The values of k_i_ were calculated from the slope of the plot between Q_t_ against t^½^ as clear from Fig. 8C. The correlation coefficient values R^2^ were 0.9856 and 0.9865 for the uptake of CR and CV dyes by DWTS adsorbent, respectively.

The Elovich model stands out as the most intriguing framework for elucidating activated chemisorption^34^, The linear expression for the Elovich model was expressed as:

6 $$Qt = {\ss}\ln \left( {\alpha \beta } \right) - \ln t$$

where α (mg/g.min) and β (g/mg) represent fixed values. The endowment α represents the initial adsorption rate (mg/g.min), while β pertains to the degree of surface coverage and the activation energy associated with chemisorption. The plot of lnt versus qt for the Elovich model is illustrated in Fig. 8D. The values of α and β were determined from the intercept and are presented in Table 4. The correlation coefficient R^2^ values were 0.9349 and 0.9441 for the uptake of CV and CR dyes by the DWTS adsorbent, respectively, Similar behavior was observed by^58^.

**Table 4:** Kinetic model parameters of pseudo-first-order, pseudo-second-order, Elovich and intra-particle diffusion for the adsorption of 50 ml / 50 Ppm CV and CR by 2 Gm DWTS at 25 °C.

Adsorption kinetic models	CV 50 ppm	CR 50 ppm
First order kinetic parameter		
Q_e_	11.49	7.309
K_1_	− 0.0394	0.03178
R^2^	0.9179	0.971
Second order kinetic parameter		
Q_e_	5.015	3.6088
K_2_	0.0012	0.0034
R^2^	0.8837	0.9414
Intra-particle diffusion		
C_i_	− 0.4751	− 0.2436
K_i_	0.2387	0.2171
R^2^	0.9856	0.9865
Elovich		
β	0.785	0.7174
α	− 1.6751	− 1.3489
R^2^	0.9349	0.9441

### Adsorption isotherm

**Fig. 9:**
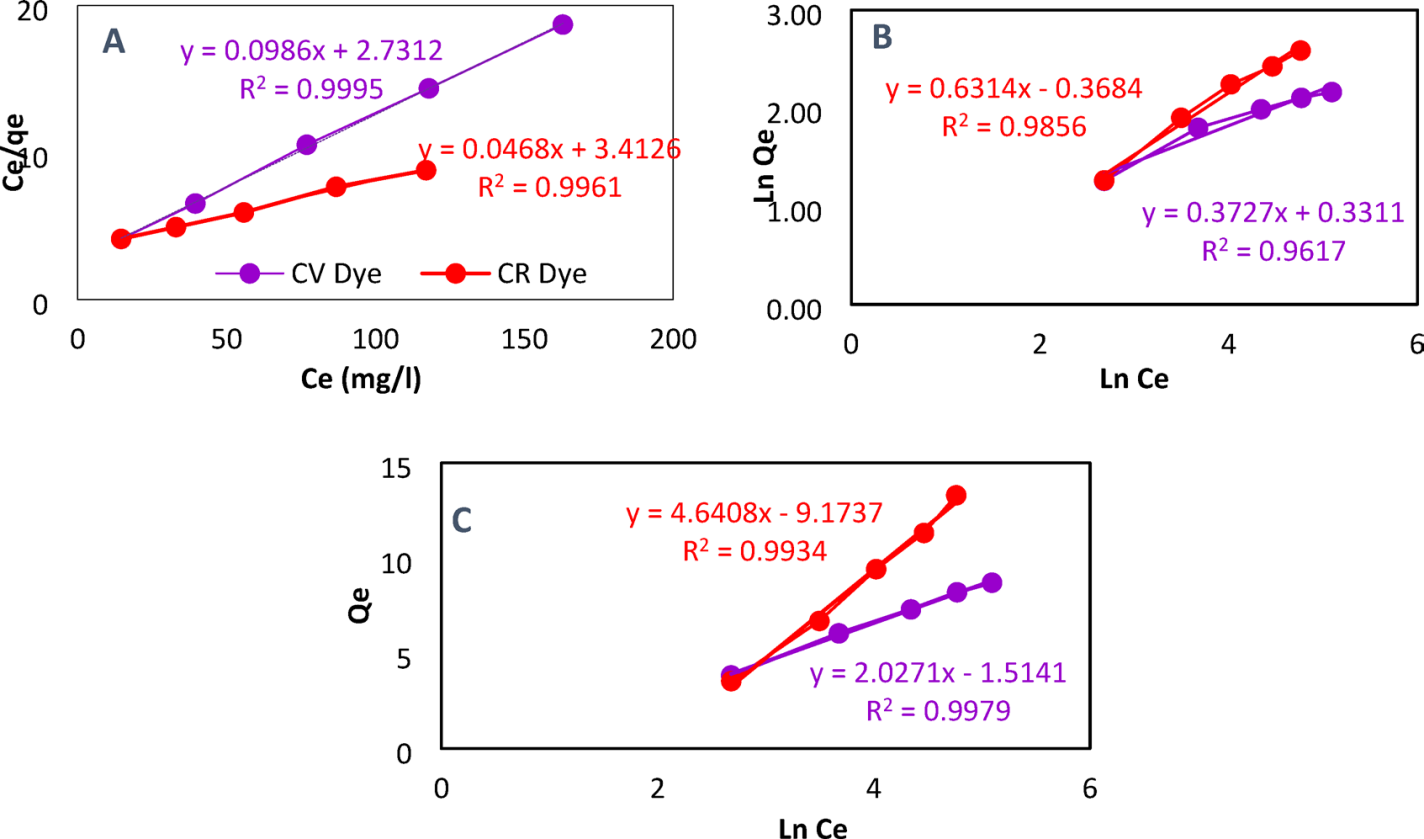
The adsorption isotherm for the Langmuir model (**A**), Freundlich model (**B**), and Temkin model (**C**) concerning the adsorption of CV and CR dye solutions utilizing DWTS adsorbent.

Adsorption isotherm characterizes the adsorbent and adsorbate interactions and helps predict the highest adsorption capacity^59,60^. The relationship between qe and Ce for the adsorption isotherms of CR and CV dyes on DWTS adsorbent was revealed from Fig. 9. In addition, Table 5 showed the data for adsorption isotherm for CR and CV dyes at varying starting concentrations (50–250 mg/L), 120 min, 2 g DWTS adsorbent, pH 9 and pH 5, respectively. The equilibrium adsorption increases notably as the initial concentration rises, and the empirical results were analyzed using three isotherm models: Temkin, Freundlich, and Langmuir. The correlation coefficient (R^2^) is used to select the proper model that describes best the adsorption process^34^.

The Langmuir isotherm model suggests that adsorption occurs in a monolayer fashion, where the adsorbate adheres to a uniform surface that features energetically equivalent binding sites^61^. The mathematical model of Langmuir isotherm is provided as following^34^:

7 $$Ce/qe = 1/qmKL + Ce/qm$$

where qe represents the amounts of CV and CR adsorbed at equilibrium (mg/g), KL is the Langmuir constant (l/mg), qm denotes the maximum monolayer coverage capacities (mg/g), and Ce indicates the equilibrium concentration of CV and CR (mg/l). By analyzing the correlation coefficients (R2), we can compare how well the isotherm equations work. The fundamental features of the Langmuir isotherm can be articulated through the dimensionless equilibrium parameter RL = 1/(1 + bCo), where Co denotes the initial dye concentration (mg/l) and b represents the Langmuir constant. The value of RL signifies the classification of the isotherm as either unfavourable (RL > 1), linear (RL = 1), favourable (0 < RL < 1), or irreversible (RL = 0)^62^.

Plotting C_e_/Q_e_ Vs C_e_, the value of K_L_ and q_m_ were calculated from the slope and intercept and presented in Table 5. The correlation coefficients (R^2^) values of Langmuir isotherm for the removal of CV and CR were 0.9995 and 0.9961, respectively as observes in Fig. 9A, Similar behavior was observed by^63^.

When applied to heterogeneous adsorption sites, the Freundlich model provides support for multilayer adsorption and is presented as^64^:

8 $$\ln qe = \ln KF + 1/nF\ln ce$$

In this context, qe represents the amount of CV and CR adsorbed at equilibrium (mg/g); Ce denotes the equilibrium concentration of CV and CR (mg/l); while KF and nF are the Freundlich constants that correspond to the adsorption capacity and intensity, respectively. In this context, KF represents the Freundlich isotherm constant (L/mg), which indicates the bonding energy. The term 1/n signifies the adsorption intensity, also referred to as the heterogeneity factor, while n denotes the degree of deviation from adsorption linearity. The values of KF and 1/nF were derived from the intercepts (ln KF) and slopes (1/nF) of the linear plots of ln qe versus ln Ce. The slope 1/nF, indicates surface heterogeneity or adsorption strength and runs from 0 to 1. The value of 1/nF approaches 0 as the adsorbent surface grows more and more diverse. Conversely, a value of 1/nF below one suggests a typical Langmuir isotherm, while a value above one indicates cooperative adsorption.

Freundlich parameters are defined from ln q_e_ versus ln C_e_ plot as shown in Fig. 9B, similar behavior observed by^30,65^.

The Freundlich exponent (n) has a decisive effect on the profile of the adsorption isotherm. In water treatment, it aims to obtain very low equilibrium concentrations which stands for the maximum permitted adsorbate concentration in the treated water. Values of (n) describe how well CV and CR can be adsorbed by DWTS adsorbent. Where: The smaller the Freundlich exponent (n), the better the CV and CR can be adsorbed, linear adsorption is found at *n* = 1; chemisorption takes place if *n* < 1 while, if the Freundlich exponent exceeds 1, the adsorption tends to physisorption process^66^.

In the adsorption process of CR and CV dyes solution on fine DWTS adsorbent particles, the Freundlich exponent (n) is 2.25 which refers to the physisorption process. The calculated adsorption intensity (n_F_) from the Freundlich model was 2.6961 and 1.5837 (1/n_F_ = 0.3709 and 0.6314) for CV and CR, respectively indicating strong interaction between adsorbate and adsorbent^60^.

Temkin isotherm model suggests a linear decrease in the amount of removal heat of all molecules with increasing coverage due to the interactions of CV, CR dye molecules with DWTS adsorbent surface-active sites, Fig. 9C describes the adsorbent-adsorbate species interaction. The model supposes that the heat of sorption of adsorbate species in the layer would decrease linearly with coverage, the Temkin model can be expressed as^64^:

9 $$Q = B\ln Kt + B\ln C_{e}$$

Disregarding the very low and high concentrations, the removal process exhibits a consistent distribution of binding energies. The B constant relates to the heat of sorption (B = RT/b; J/mol), K_t_ (L/mg) denotes the equilibrium binding constant, and B signifies the Temkin sorption isotherm constant.

The values for the Temkin isotherm (Kt and B) were derived from the intercept and slope of the graph plotting Qe against ln Ce, and these results are presented in Table 5. The correlation coefficient values, R² = 0.9993 and 0.9934 for CV and CR uptake by DWTS adsorbent are clearly illustrated in Fig. 9C^30,34^.

**Table 5:** Isotherm models’ parameters for adsorption of 50 ml/50 ppm CR and CV dyes onto 2 gm DWTS adsorbent at25 °C.

Isotherm model	CV dye	CR dye
Langmuir		
q_m_ (mg ^−1^ calculated)	10.1419	21.368
K_L_	0.0361	0.0137
R^2^	0.9995	0.9961
Freundlich		
K_F_ (mg g^−1^) × (l mg^−1^)1/n	1.40382	1.4454
N_f_	2.6831	1.5837
R^2^	0.9617	0.9856
Temkin		
B (l mg^−1^)	7.592	103.627
K_T_ (KJ mol^−1^)	0.8192	0.9153
R^2^	0.9979	0.9934

### Optimization and validation

To achieve the highest efficiency in dye removal, it is essential to evaluate the optimal adsorption conditions namely contact time, adsorbent dose, pH, and dye concentration for CV and CR dyes on the DWTS adsorbent in a batch mode, ensuring the accuracy and validity of these optimal conditions. The parameters outlined below were established to achieve the targeted CV and CR removal efficiency: The pH was maintained at 9 and 5 for CV and CR dyes, respectively. The concentrations of CV and CR were set to 50 mg/L, the dose of DWTS adsorbent was 2 gm, and the stirring time was established at 120 min. The parameters facilitated a removal efficiency of 86.9% for CV and 94.3% for CR, respectively.

### Comparison of maximum adsorption capacity with another adsorbent

Table 6 shows the results of a comparison between the claimed DWTS adsorbent and other adsorbents for the removal of CV and CR dyes. It is clear that DWTS demonstrated a strong ability to adsorb dyes effectively, especially when compared to other materials that are either costly or necessitate extended chemical or thermal processes, which restrict their practical use. Nonetheless, numerous operational conditions influence the variations in adsorption capacities, including volume, pH, dosage, concentration, time, and more. Based on the evidence presented, the proposed adsorbent demonstrates both efficiency and practical, economic viability.

**Table 6:** Analysis of the maximum adsorption capacity in the current study relative to previously documented findings.

Adsorbent	Removal efficiency (%)	Max. adsorption (mg/g)	Refs.
CV dye			
Rice husk ash (RHA)	53.6%	8.3	^67^
Carbon embedded zeolite from rice husk (Z-RHA)	96.45%	19.28	^67^
Pine cones	99%	17.8	^68^
Carbon embedded silica from rice husk (CES)	90.69%	18.78	^67^
Nascent rice husk	57.40–98.19%	24.48	^63^
DWTS adsorbent	88.6%	10.14	This study
CR dye			
Leaves powder of Grewia asiatica (falsa)	77–99%	18.167	^69^
Corn cons	92.5–98.6%	37.81	^70^
Mango leaves	90%	21.28	^71^
Breadfruit leaf biochar	99.9	17.8	^58^
Kenaf-based activated carbon	95	14.2	^72^
Cotton wood	97.08%	45.97	^73^
DWTS adsorbent	94.34%	21.37	This study

### Adsorption mechanism

The proposed adsorption mechanism (Fig. 8) plays a crucial role in comprehending the adsorption process. One of the most important factors in adsorption is electrostatic attraction, as shown by the accelerated removal of CV under basic circumstances (pH > Pzc), which exhibited stronger interactions with negatively charged functional groups on DWTS, as evident from the pronounced spectral changes in FTIR and the reduction in mesoporous volume in BET analysis. For adsorption of CR onto the DWTS, according to the FTIR data, formed hydrogen bonds and electrostatic interactions with protonated groups, favoring adsorption at acidic pH levels, the smaller size allowed to access micropores, as indicated by the changes in microporous volume^69^. Thus, both FTIR and BET analyses demonstrated that DWTS effectively absorbs both CV and CR dyes through distinct mechanisms involving electrostatic interactions, hydrogen bonding, and pore occupation. The kinetic study indicates that the intra-particle diffusion model is the most suitable for the processes examined. As shown in Fig. 8, the plot does not intersect the origin, leading to the conclusion that intra-particle diffusion is more significant than film diffusion^74,75^.

The hydroxyl groups present on the DWTS adsorbent surface have the capacity to either gain or lose a proton, resulting in a variation in the surface charge that shifts in response to changing pH values. At low pH, the surface functional groups become protonated, resulting in the formation of a positive charge on the surface. With an increase in pH, there is a reduction in the protonation of the –OH and –COOH groups located on the surface of the DWTS adsorbent, leading to an enhanced attraction between the dye and the DWTS. Carboxylate groups exhibited a positive charge, leading to electrostatic interactions between the positively charged CV and the positively charged adsorbent surface^76^. Therefore, intra-particle diffusion of CR and CV molecules occurs in the porous structure of DWTS adsorbent, where hydrogen bonds (also known as Van der Waals or electrostatic attraction forces) are formed between the molecules and the hydroxyl groups on the DWTS adsorbent surface until the process reaches equilibrium.

### Disposal of the used adsorbent

The disposal of dye-loaded adsorbents requires careful consideration to prevent potential secondary pollution effects. The proper disposal of spent adsorbents highlights the necessity for more sustainable processes that contribute to environmental preservation.

It is essential to effectively eliminate waste adsorbents through methods such as landfilling, combustion for energy recovery, or dissolution in strong acids or bases. It can also be integrated into construction materials, in the production of porous fired clay bricks^77^, and the manufacturing of pottery for use in making bricks^78^. The production of refractory bricks is proposed as a viable option for the secure disposal of spent adsorbents, enhancing their practical use. The utilization of dried wastewater treatment sludge (DWTS) as a component in cement-based materials presents a viable strategy for enhancing the overall usage and mitigating the environmental impact. DWTS is characterized by a high content of silica-alumina phases, which demonstrate pozzolanic activity following processes of drying, grinding, and calcination. This attribute endows DWTS with properties comparable to those of traditional supplementary cementitious materials. Modifications to the sludge production process and variation in coagulant types can significantly influence the physical and chemical characteristics of DWTS. Incorporating small quantities of DWTS into cement mixtures can lead to the formation of additional hydration products and the refinement of pore structures within the cement matrix. These changes are instrumental in enhancing both the mechanical properties and durability of the resultant cement-based samples. The application of high-volume DWTS is also promising for the fabrication of artificial aggregates, lightweight concrete, and sintered bricks. Moreover, the composition of calcined DWTS bears resemblance to that of clay, thereby positioning it as a potential raw material for the production of cement clinker. Furthermore, cement-based materials have demonstrated effectiveness in immobilizing heavy metal ions present in DWTS. The implementation of alkali-activated binders, magnesium-based cement, and carbon curing technologies can further minimize the risks associated with heavy metal leaching, thereby enhancing the sustainability and safety of using DWTS in construction applications^79–83^.

### Reusability and performance of DWTS in future work

**Fig. 10:**
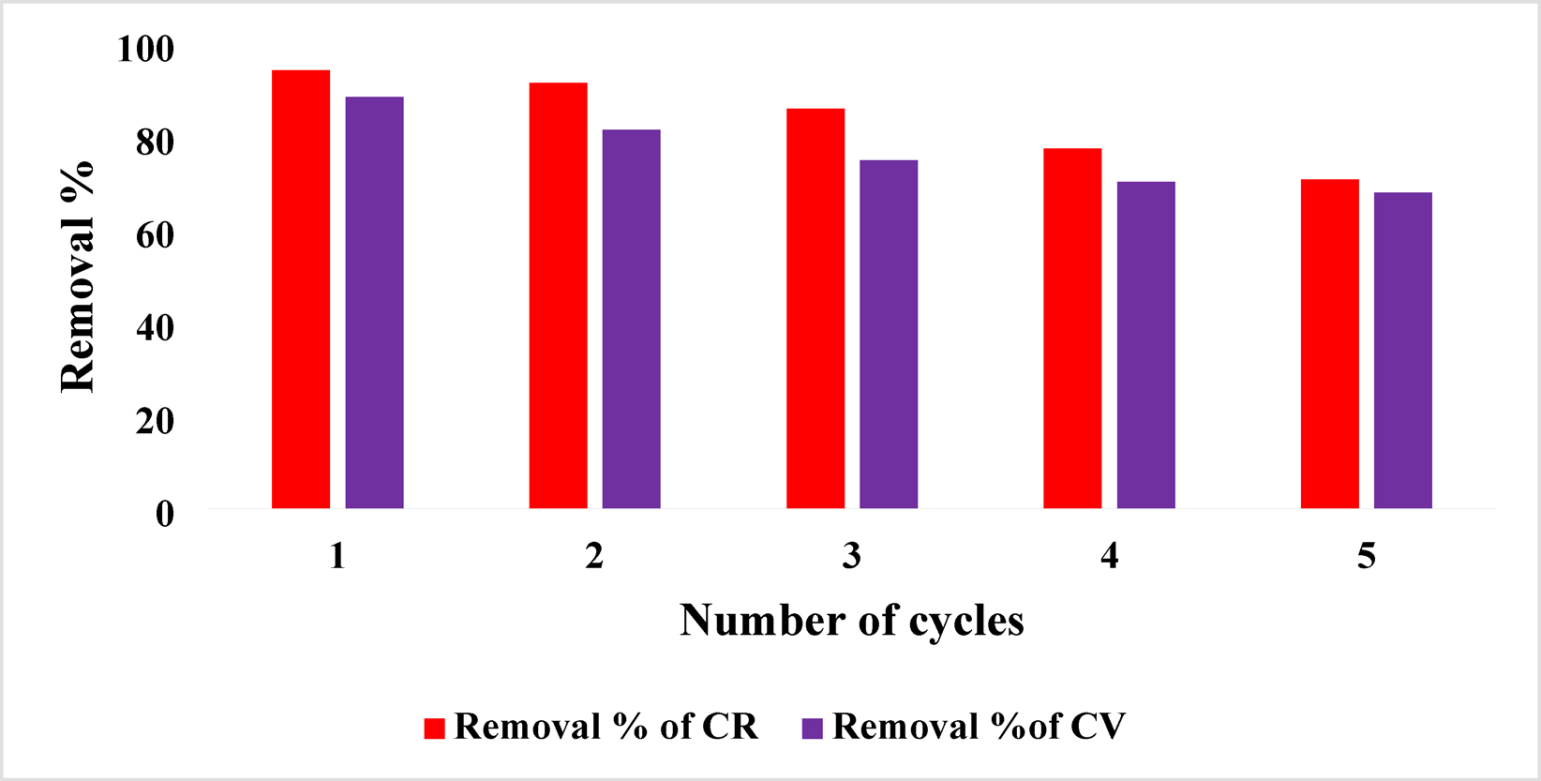
Removal efficiency of crystal violet (CV) and congo red (CR) with regeneration cycles in dye wastewater treatment systems.

To evaluate the reusability of DWTS, we commenced the experiments by 2 g of the sludge, allowing it to achieve equilibrium adsorption with 50 ml of dye solutions (50 ppm) for both crystal violet (CV) and Congo red (CR). The efficacy of various eluting agents (HCl, HNO_3_, NaOH, ethanol, and 50% mixture of acetic acid (0.1 M) and methanol) was assessed at different ratios to recover the dyes from DWTS. The 50% mixture of acetic acid and methanol emerged as the most effective eluting agent, achieving satisfactory recovery rates for both CV and CR. Following regeneration cycles by treating the DWTS under the same conditions to evaluate the adsorption capacity post-recovery. It was observed that the removal percentages of both dyes by DWTS remained relatively high (Fig. 10). However, some challenges arose during the desorption process, as desorption rate for CV was below 70%, due to strong interactions such as cation exchange and π-π stacking^84^. Conversely, CR yielded a higher desorption rate of around 80%, albeit with concerns about residue retention. Additionally, it was noted that repeated acid/alkali treatments impacted the structural integrity and porosity of the DWTS, which may compromise its adsorption capacity in subsequent cycles. Thus, while the DWTS demonstrates potential for reusability, structural integrity and dye-specific interactions must be carefully managed to optimize adsorption capacity over multiple cycles regarding sustainability and potential modifications to enhance the performance of DWTS in future work^85,86^.

This study acknowledges several limitations that impact the interpretation of the findings and their implications. First, the efficacy of dried wastewater treatment sludge (DWTS) in adsorbing dyes is likely influenced by the variability in wastewater composition, as complex mixtures or interfering substances may hinder the adsorption process a factor not thoroughly explored in this research. Additionally, the initial concentrations of Crystal Violet (CV) and Congo Red (CR) dyes used may not fully represent the range encountered in real-world applications, with higher concentrations potentially leading to saturation of the adsorbent and reducing its effectiveness; thus, a more comprehensive examination of varied dye concentrations is necessary. Furthermore, the study lacks an in-depth analysis of the thermal stability of DWTS and the potential leaching of metals such as aluminum and iron, which could negatively affect water quality, emphasizing the need for future investigations to incorporate Thermal Gravimetric Analysis (TGA). It is worth noting that the treated wastewater containing dyes is not directly reused as it is drained to the sewage network for biological treatment.Additionally, adjusting the pH could enhance the precipitation and removal of such harmful elements from the treated water. Addressing these limitations in subsequent research will improve the practical applications and understanding of DWTS as a promising adsorbent for wastewater treatment.

### Conclusion

This research highlights the pressing global challenges of water scarcity and pollution, particularly due to the widespread use of synthetic dyes in various industries. The study focused on the removal of two common dyes, Crystal Violet and Congo Red from wastewater usingDrinking Water Treatment Sludge (DWTS) as an eco-friendly and cost-effective adsorbent. The findings demonstrate that DWTS, when thermally treated, exhibits significant potential for dye adsorption, making it a viable alternative to traditional methods. The optimization of adsorption conditions revealed that factors such as contact time, initial dye concentration, pH, and adsorbent dosage play crucial roles in the efficiency of dye removal. The maximum removal efficiencies achieved were 88.6% for CV and 94.34% for CR, indicating the effectiveness of DWTS in treating dye-contaminated water. Kinetic and isotherm studies indicated a contribution of physical and chemical adsorption process.

Characterization of the DWTS adsorbent revealed its mesoporous structure and the presence of functional groups that facilitate dye interaction. The study also emphasizes the importance of utilizing waste materials like DWTS in line with circular economy principles, reducing landfill waste while providing a sustainable solution for water treatment. In conclusion, the research underscores the potential of DWTS as an effective and sustainable adsorbent for the removal of harmful dyes from wastewater, contributing to the broader efforts in addressing water pollution and promoting environmental sustainability. Looking ahead, future research should focus on scaling up the application of DWTS in real-world wastewater treatment scenarios, exploring its effectiveness across various industrial effluents. Additionally, investigations into the long-term stability and reusability of DWTS as an adsorbent, along with the development of protocols for the safe disposal or repurposing of spent adsorbents, are essential. The integration of DWTS into current wastewater treatment systems, along with its use in various environmental remediation efforts, holds great promise for enhancing circular economy principles and fostering sustainable waste management practices.

## Data Availability

The data presented in this study are available on request to the corresponding author.
